# Prediction of 1-year clinical outcomes using the SYNTAX score in patients with prior heart failure undergoing percutaneous coronary intervention: sub-analysis of the SHINANO registry

**DOI:** 10.1007/s00380-016-0896-9

**Published:** 2016-10-05

**Authors:** Masatoshi Minamisawa, Takashi Miura, Hirohiko Motoki, Hideki Kobayashi, Masanori Kobayashi, Hiroyuki Nakajima, Hikaru Kimura, Hiroshi Akanuma, Eiichiro Mawatari, Toshio Sato, Shoji Hotta, Yuichi Kamiyoshi, Takuya Maruyama, Noboru Watanabe, Takayuki Eisawa, Shinichi Aso, Shinichiro Uchikawa, Keisuke Senda, Takehiro Morita, Naoto Hashizume, Naoyuki Abe, Soichiro Ebisawa, Atsushi Izawa, Yusuke Miyashita, Jun Koyama, Uichi Ikeda, On behalf of the SHINANO Registry Investigators

**Affiliations:** 1 Department of Cardiovascular Medicine, Shinshu University School of Medicine, -1-1 Asahi, Matsumoto, Nagano 390-8621 Japan; 2 Department of Cardiology, Shinshu Ueda Medical Center, Ueda, Japan; 3 Department of Cardiology, Matsumoto Kyoritsu Hospital, Matsumoto, Japan; 4 Department of Cardiology, Nagano Matsushiro General Hospital, Nagano, Japan; 5 Department of Cardiology, Saku Central Hospital, Saku, Japan; 6 Department of Cardiology, Iida Municipal Hospital, Iida, Japan; 7 Department of Cardiology, Kita Alps Medical Center, Azumino, Japan; 8 Department of Cardiology, Shinonoi General Hospital, Nagano, Japan; 9 Department of Cardiology, Ina Central Hospital, Ina, Japan; 10 Department of Cardiology, Aizawa Hospital, Matsumoto, Japan; 11 Department of Cardiology, Hokushin General Hospital, Nakano, Japan; 12 Department of Cardiology, Komoro Kosei General Hospital, Komoro, Japan; 13 Department of Cardiology, Azumino Red Cross Hospital, Azumino, Japan; 14 Department of Cardiology, Okaya Municipal Hospital, Okaya, Japan

**Keywords:** Coronary artery disease, Heart failure, SYNTAX score, Prognosis

## Abstract

Although coronary artery disease (CAD) is common in patients with heart failure (HF), little is known about the prognostic significance of coronary lesion complexity in patients with prior HF undergoing percutaneous coronary intervention (PCI). The aim of this study was to investigate whether the coronary Synergy between Percutaneous Coronary Intervention with TAXus and Cardiac Surgery (SYNTAX) score could improve risk stratification in HF patients with CAD. Two hundred patients (mean age 73 ± 11 years, left ventricular ejection fraction 49 ± 15 %) with prior HF who underwent PCI were divided into two groups stratified by SYNTAX score (median value 12) and tracked prospectively for 1 year. The study endpoint was the composite of major adverse cardiovascular events (MACE), including all-cause death, myocardial infarction, stroke, and hospitalization for worsening HF. Adverse events were observed in 39 patients (19.5 %). Patients with high SYNTAX scores (*n* = 100) showed worse prognoses than those with low scores (*n* = 100) (26.0 vs. 13.0 %, respectively, *P* = 0.021). In multivariate Cox-regression analysis, SYNTAX score ≥12 was significantly associated with MACE (hazard ratio: 1.99, 95 % confidence interval: 1.02–3.97; *P* = 0.045). In patients with prior HF and CAD, high SYNTAX scores predicted a high incidence of MACE. These results suggest that the SYNTAX score might be a useful parameter for improving risk stratification in these patients.

## Introduction

Heart failure (HF) is a serious healthcare problem in today’s aging society. Despite significant advances in the treatment of chronic HF, the disease tends to follow a progressive course with high mortality and morbidity rates [1–3]. Patients with HF are at significant risk for recurrent cardiovascular events such as death, myocardial infarction (MI), stroke, and hospitalization for worsening HF. Therefore, the secondary prevention of cardiovascular events is invaluable for improving the prognostic outlook of HF patients. A novel risk stratification system would provide critical information that could result in more aggressive therapy and lead to improved patient survival. Coronary artery disease (CAD) has contributed to the increased prevalence of HF and is associated with cardiovascular events in patients with HF [4–7]. The Synergy between Percutaneous Coronary Intervention with TAXus and Cardiac Surgery (SYNTAX) score, a measure of coronary lesion complexity, has been proposed for use in the risk stratification of patients with untreated left main trunk or 3-vessel CAD [8, 9]. However, the prognostic significance of the SYNTAX score for risk stratification in HF patients is poorly understood. We hypothesized that the SYNTAX score would predict adverse cardiovascular events in patients with HF. The aim of this study was to investigate whether the coronary SYNTAX score could improve risk stratification in HF patients with CAD.

## Materials and methods

### Study population

This cohort study retrospectively reviewed data available from the SHINANO registry (Shinshu prospective multi-center study of elderly patients with CAD undergoing percutaneous coronary intervention (PCI)) obtained between August 2012 and July 2013. A detailed summary of the methods and design of this registry has been published previously [10]. Briefly, the SHINANO registry is a prospective, multi-center observational registry of patients with any CAD diagnosis, including stable angina, ST-segment elevation MI, non-ST-segment elevation MI, and unstable angina, undergoing PCI at 16 collaborating hospitals located in the Nagano prefecture, Japan. As it is an all-comer registry, there are no exclusion criteria. The institutional review board approved the protocol, which was registered at the University Hospital Medical Information Network (UMIN000010070), and informed consent was obtained from each patient before enrollment. This study was performed in accordance with the Declaration of Helsinki.

Among the 1923 patients registered in the SHINANO registry, we screened 254 patients with a history of HF. After excluding patients with a history of coronary artery bypass grafting (CABG), as well as those with missing data concerning left ventricular ejection fraction (LVEF) or no SYNTAX score, we enrolled 200 patients with prior HF into the final study. All patients were prospectively followed for 12 months from the date of the PCI procedure. The study endpoint was the composite of major adverse cardiovascular events (MACE), including all-cause death, MI, stroke, and hospitalization for worsening HF using a time-to-first-event analysis.

### Study definitions

MI was diagnosed according to the American College of Cardiology/American Heart Association (ACC/AHA) guidelines [11]. Stroke was defined as an ischemic cerebrovascular event that persisted for ≥24 h and was diagnosed by a neurologist [12]. Prior HF was based on a previous diagnosis of HF according to the Framingham criteria [13] or a history of hospitalization for worsening HF. Body mass index was defined as weight in kilograms divided by the square of height in meters. Patients with systolic blood pressure >140 mmHg and/or diastolic pressure >90 mmHg and those taking anti-hypertensive agents were considered to have hypertension. Dyslipidemia was defined as a serum total cholesterol concentration ≥220 mg/dL, low-density lipoprotein cholesterol ≥140 mg/dL, or the need for treatment with lipid-lowering agents. Diabetes mellitus was defined as hemoglobin (Hb) A1c ≥6.5 %, random plasma glucose ≥220 mg/dL, or a clinical history of oral hypoglycemic agent and/or insulin use. Patients were considered smokers if they were current smokers. CAD was defined as >50 % stenosis in a coronary vessel on angiography, history of CABG or PCI, or history of MI. Multi-vessel disease was defined as the presence of a ≥75 % lesion in ≥2 major coronary arteries. The SYNTAX score was calculated as previously described [9]. Complete revascularization was considered to have occurred when all stenotic main vessels and all side branches greater than 2 mm in diameter were revascularized [14]. Estimated glomerular filtration rate (eGFR) was calculated using the Modification of Diet and Renal Disease equation with coefficients modified for Japanese patients [15]. Chronic kidney disease was defined as an eGFR <60 ml/min/1.73 m^2^. LVEF was calculated using the Teichholz method in patients without regional wall motion abnormality or the biplane Simpson’s method in those with regional wall motion abnormality [16, 17]. All PCI procedures and selection of medical treatments after PCI were at the discretion of the treating physician.

### Statistical analysis

Continuous variables were summarized as mean ± standard deviation if normally distributed or as median and interquartile range otherwise. Normality was evaluated using the Shapiro–Wilk *W* test. Comparisons of baseline categorical data between the two groups were analyzed using two-sided Chi-squared tests, whereas differences between continuous variables were analyzed using an unpaired *t* test or the Mann–Whitney *U* test. The optimal cutoff value for MACE prediction was chosen as the value that maximized sensitivity and specificity on the receiver operating characteristics (ROC) curve. Kaplan–Meier curves were constructed from the date of the PCI procedure to the MACE and were compared using the log-rank test. Cox proportional hazards regression analysis was performed to identify the MACE predictors, using variables that included clinical characteristics and risk factors. Multivariate analysis was performed using all variables with a *P* value <0.1 in the univariate analysis. A *P* value <0.05 was considered to indicate statistical significance. All analyses were performed using SPSS version 22.0 (SPSS, Chicago, IL, USA).

## Results

### Patient characteristics

**Fig. 1:**
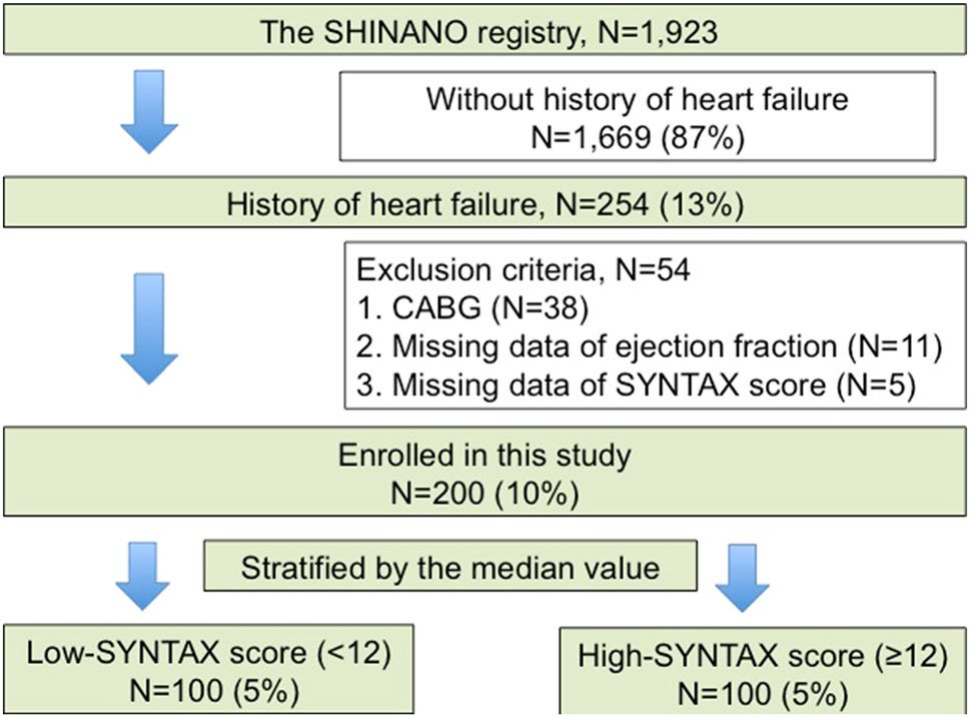
Study design. *CABG* coronary artery bypass graft

All of the patients initially enrolled in the study completed the follow-up. Using the median value of the SYNTAX score (12), patients were divided into a high-SYNTAX score group (*n* = 100) and a low-SYNTAX group (*n* = 100) (Fig. 1). During the 12-month follow-up period, adverse events were observed in 39 patients (19.5 %) and included deaths (*n* = 25), MIs (*n* = 5), strokes (*n* = 5), and hospitalizations for HF (*n* = 18) (Table 1). The baseline clinical characteristics are listed in Table 2. Patients with high SYNTAX scores did not differ from those with low scores with respect to age, sex, the prevalence of hypertension, dyslipidemia, diabetes mellitus, atrial fibrillation, chronic kidney disease, prior stroke, hemodialysis, or peripheral artery disease. Patients with high SYNTAX scores had significantly more severe HF symptoms, as estimated by New York Heart Association (NYHA) functional class, compared with those with low SYNTAX scores. As expected, the prevalence of multi-vessel disease was significantly higher in patients with high SYNTAX scores compared with those with low scores. Complete revascularization was significantly rarer in patients with high SYNTAX scores. The use of angiotensin-converting enzyme inhibitors, angiotensin receptor blockers, beta-blockers, and statins was similar between the two groups. Hb, low-density lipoprotein cholesterol, eGFR, and HbA1c levels were similar between the two groups, whereas levels of brain natriuretic peptide tended to be higher in patients with high SYNTAX scores than in those with low scores. LVEF was significantly lower in patients with high SYNTAX scores than in those with low scores.

**Table 1:** Clinical outcomes

	Overall (*n* = 200)	Low-SYNTAX group (<12) (*n* = 100)	High-SYNTAX group (≥12) (*n* = 100)	*P* value
MACE, *n* (%)	39 (19.5)	13 (13.0)	26 (26.0)	0.021
All-cause death, *n* (%)	25 (12.5)	4 (4.0)	21 (21.0)	<0.001
Cardiac death, *n* (%)	13 (6.5)	1 (1.0)	12 (12.0)	0.001
Myocardial infarction, *n* (%)	5 (2.5)	3 (3.0)	2 (2.0)	0.58
Stroke, *n* (%)	5 (2.5)	2 (2.0)	3 (3.0)	0.60
Hospitalization for heart failure, *n* (%)	18 (9.0)	7 (7.0)	11 (11.0)	0.24

Values are number (%)

*MACE* major adverse cardiac events (including all-cause death, myocardial infarction, stroke, and hospitalization for heart failure)

**Table 2:** Baseline characteristics of patients according to SYNTAX score

	Overall (*n* = 200)	Low-SYNTAX group (<12) (*n* = 100)	High-SYNTAX group (≥12) (*n* = 100)	*P* value
Age	73 ± 11	73 ± 11	74 ± 11	0.58
Female sex, *n* (%)	47 (23.4)	18 (18.0)	29 (29.0)	0.067
Body mass index (kg/m^2^)	22.6 ± 4.1	23.1 ± 3.9	22.2 ± 4.3	0.14
Ischemic etiology (%)	66.7	58.3	75.6	0.001
NYHA functional class ≥III, *n* (%)	60 (30.0)	23 (23.0)	37 (37.0)	0.031
Comorbidities				
Hypertension, *n* (%)	157 (78.1)	78 (78.0)	79 (79.0)	0.86
Dyslipidemia, *n* (%)	127 (63.2)	62 (62.0)	65 (65.0)	0.66
Diabetes mellitus, *n* (%)	76 (37.8)	40 (40.0)	36 (36.0)	0.56
Current smoker, *n* (%)	25 (12.4)	12 (12.0)	13 (13.0)	0.87
Atrial fibrillation, *n* (%)	56 (28.0)	34 (34.0)	22 (22.0)	0.059
Chronic kidney disease, *n* (%)	132 (66.0)	72 (72.0)	60 (60.0)	0.10
Prior stroke, *n* (%)	27 (13.5)	12 (12.0)	15 (15.0)	0.68
Hemodialysis, *n* (%)	22 (11.0)	15 (15.0)	7 (7.0)	0.11
Peripheral artery disease, *n* (%)	37 (18.5)	17 (17.0)	20 (20.0)	0.72
Angiographic data				
Target coronary lesion				
Right coronary artery, *n* (%)	72 (36.0)	41 (41.0)	31 (31.0)	0.19
Left anterior descending artery, *n* (%)	92 (46.0)	40 (40.0)	52 (52.0)	0.12
Left circumflex artery, *n* (%)	31 (15.5)	18 (18.0)	13 (13.0)	0.44
Left main trunk, *n* (%)	5 (2.5)	1 (1.0)	4 (4.0)	0.37
De novo lesion, *n* (%)	170 (67.5)	83 (83.0)	87 (87.0)	0.55
Only POBA	41 (20.5)	21 (21.0)	20 (20.0)	0.86
Type of implanted stent				
Drug-eluting stent, *n* (%)	135 (67.5)	69 (69.0)	66 (66.0)	0.76
Bare metal stent, *n* (%)	24 (12.0)	10 (10.0)	14 (14.0)	0.52
Calcification lesion, *n* (%)	73 (36.5)	32 (32.0)	41 (41.0)	0.24
Bifurcation lesion, *n* (%)	58 (29.0)	20 (20.0)	38 (38.0)	0.008
Ostial lesion, *n* (%)	15 (7.5)	5 (5.0)	10 (10.0)	0.28
Multi-vessel, *n* (%)	83 (41.3)	23 (23.0)	60 (60.0)	<0.001
SYNTAX score	13.7 ± 9.5	6.4 ± 2.4	21.1 ± 8.1	<0.001
Complete revascularization, *n* (%)	123 (61.5)	72 (72.0)	51 (51.0)	0.001
Acute coronary syndrome, *n* (%)	61 (30.3)	27 (27.0)	34 (34.0)	0.28
STEMI on admission, *n* (%)	38 (19.0)	10 (10.0)	28 (28.0)	0.002
Killip class IV on admission, *n* (%)	11 (5.5)	1 (1.0)	10 (10.0)	0.010
Medications at discharge				
Aspirin, *n* (%)	187 (93.5)	96 (96.0)	91 (91.0)	0.21
Thienopyridines, *n* (%)	167 (83.5)	89 (89.0)	78 (78.0)	0.049
Warfarin, *n* (%)	56 (28.0)	33 (33.0)	23 (23.0)	0.16
ACE-inhibitor/ARB, *n* (%)	157 (78.1)	81 (81.0)	76 (76.0)	0.79
Beta-blocker, *n* (%)	118 (58.7)	57 (57.0)	61 (61.0)	0.44
Statin, *n* (%)	134 (66.7)	61 (61.0)	73 (73.0)	0.053
Insulin user, *n* (%)	18 (9.0)	12 (12.0)	6 (6.0)	0.14
Laboratory data				
Hemoglobin (g/dL)	12.7 ± 3.0	12.7 ± 2.0	12.8 ± 3.9	0.84
LDL-C (mg/dL)	99.3 ± 33.8	96.0 ± 28.4	102.8 ± 38.6	0.17
eGFR (mL/min/1.73 m^2^ surface area)	50.3 ± 23.9	49.2 ± 22.5	51.3 ± 25.4	0.53
Hemoglobin A1c (%)	6.1 ± 1.0	6.1 ± 0.9	6.1 ± 1.0	0.86
BNP (pg/mL)	304 [133, 733]	241 [123, 456]	427 [137, 1021]	0.067
LV ejection fraction (%)	49.4 ± 14.7	52.6 ± 15.7	46.2 ± 13.0	0.002

Values are number (%), mean ± standard deviation, or median [25th, 75th percentiles]

*ACE* angiotensin-converting enzyme, *ARB* angiotensin receptor blocker, *BNP* B-type natriuretic peptide, *eGFR* estimated glomerular filtration rate, *LDL* low-density lipoprotein cholesterol, *LV* left ventricular, *MACE* major adverse cardiac events (including all-cause death, myocardial infarction, stroke, and hospitalization for heart failure), *POBA* plain old balloon angioplasty

### Predictors of MACE

**Fig. 2:**
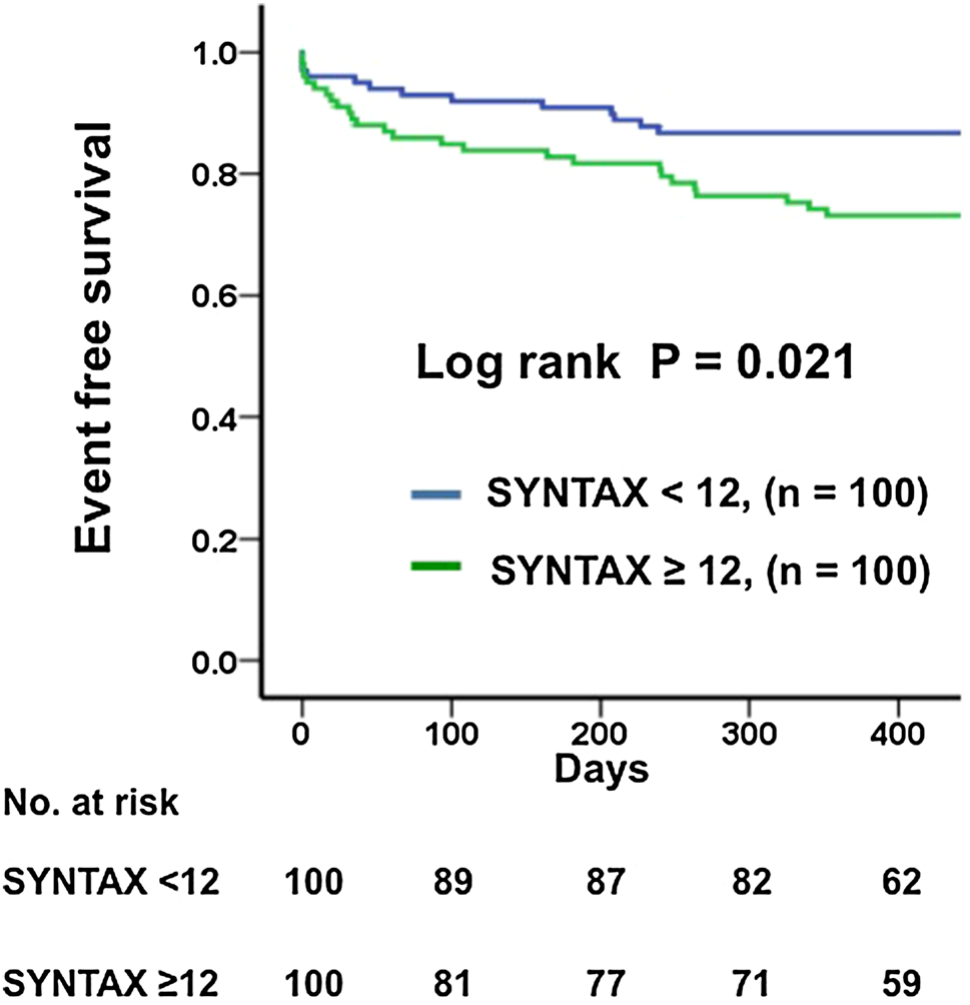
Kaplan–Meier curves for MACE according to SYNTAX score. *MACE* major adverse cardiac events (including all-cause death, myocardial infarction, stroke, and hospitalization for HF)

**Fig. 3:**
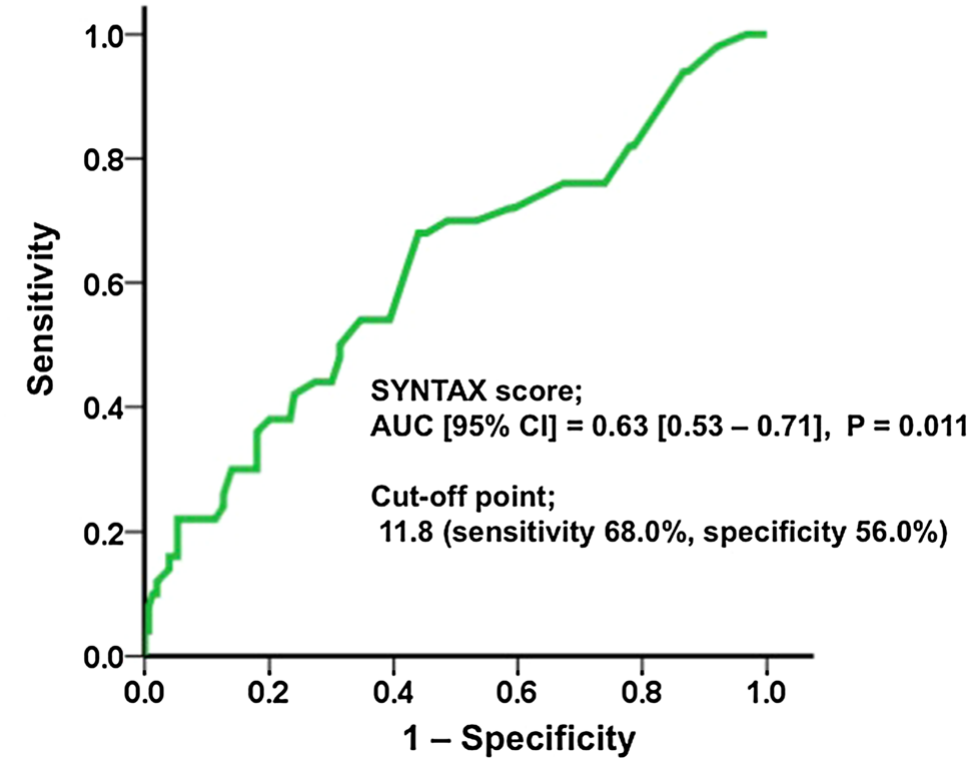
Receiver-operating characteristic (ROC) curve for predicting adverse events. The area under the ROC curve (AUC) for the SYNTAX score was 0.63, with an optimal ROC cutoff point of 11.8. *CI* confidence interval

In the Kaplan–Meier analysis, patients with high SYNTAX scores (≥12) showed worse prognoses than those with low SYNTAX scores (<12) (26.0 vs. 13.0 %, respectively, *P* = 0.021) (Fig. 2). After multivariate Cox proportional hazards analysis, which included age, sex, and all variables with *P* < 0.1 in the univariate analysis, a high SYNTAX score predicted a poor prognosis (Table 3). Based on the SYNTAX scores in our study, the area under the ROC curve (AUC) was 0.63 (Fig. 3) and the optimal cutoff point for predicting adverse events was a SYNTAX score of 11.8 (sensitivity 68.0 %, specificity 56.0 %).

**Table 3:** Cox Proportional Hazards Analyses of MACE

	Univariate		Multivariate*	
	HR (95 % CI)	*P* value	HR (95 % CI)	*P* value
Age	1.03 (1.02–1.10)	0.087	1.02 (0.98–1.05)	0.36
Female sex	1.55 (0.78–3.06)	0.21	1.25 (0.62–2.52)	0.53
NYHA functional class ≥ III	1.99 (1.06–3.75)	0.033	1.87 (0.98–3.56)	0.057
Diabetes mellitus	1.66 (0.89–3.11)	0.11		
Atrial fibrillation	1.56 (0.81–3.00)	0.18		
Chronic kidney disease	1.44 (0.63–3.10)	0.35		
Hemodialysis	0.19 (0.025–1.35)	0.17		
Prior stroke	0.77 (0.27–2.16)	0.16		
Peripheral artery disease	1.24 (0.57–2.70)	0.59		
Multi-vessel disease	1.14 (0.75–1.74)	0.53		
SYNTAX score ≥12	2.14 (1.10–4.17)	0.025	1.99 (1.02–3.90)	0.045
Aspirin	0.81 (0.43–1.55)	0.53		
Thienopyridines	0.79 (0.42–1.48)	0.46		
ACE-inhibitor/ARB	1.16 (0.60–2.24)	0.65		
Beta-blocker	0.76 (0.50–1.45)	0.41		
Insulin	1.55 (0.60–4.00)	0.36		
Hemoglobin	0.97 (0.86–1.09)	0.58		
eGFR	0.99 (0.96–1.03)	0.82		
BNP	1.00 (1.00–1.01)	0.75		
LV ejection fraction	0.98 (0.96–1.00)	0.17		

*ACE* angiotensin-converting enzyme, *ARB* angiotensin receptor blocker, *BNP* B-type natriuretic peptide, *CI* confidence interval, *eGFR* estimated glomerular filtration rate, *HR* hazard ratio, *LV* left ventricular, *MACE* major adverse cardiac events (including all-cause death, myocardial infarction, stroke, and hospitalization for heart failure)

* Adjusted for age, sex, NYHA functional class ≥III, and SYNTAX score ≥12

## Discussion

To the best of our knowledge, this is the first report to investigate the prognostic significance of the SYNTAX score for predicting cardiovascular events in patients with prior HF undergoing PCI. We demonstrated that a high SYNTAX score was an independent predictor of MACE in this patient population.

The SYNTAX score established itself as an important tool in the SYNTAX trial, which pioneered the Heart Team approach, in which the interventional cardiologist and cardiac surgeon determined the optimal revascularization modality for patients with untreated left main trunk or 3-vessel CAD [9]. SYNTAX is a very convenient scoring system for assessing the coronary lesion complexity in patients with CAD. Previous studies have demonstrated that a higher score is an independent marker of poor cardiovascular prognosis in patients with CAD [18, 19]. Importantly, risk stratification using the SYNTAX score has been validated in patients with CAD [20, 21]; however, there have been no studies using the SYNTAX score for risk stratification in HF patients undergoing PCI. In our study, we demonstrated that the SYNTAX score had predictive value for MACE in prior HF patients with CAD undergoing PCI. This result was consistent with previous reports on the prognostic value of the SYNTAX score in patients with acute MI [22].

We demonstrated that the AUC of the SYNTAX score for predicting adverse events was 0.63. This value was similar to that reported by a previous study which evaluated the value of the SYNTAX score for predicting 12-month clinical outcomes in acute MI (AUC: 0.65) [23]. Furthermore, the AUC of the SYNTAX score for predicting 12-month adverse events was 0.60 in the SHNINO registry study which included prior HF and non-HF patients. This AUC also approximates that of the SYNTAX score for predicting 5-year adverse events which was reported as 0.61 in the SYNTAX trial [18]. It remains unclear whether the SYNTAX score is a more useful parameter in patients with more severe cardiovascular diseases, such as HF and MI, than in those with lone CAD. A high SYNTAX score was also an independent predictor of MACE after multivariate analysis. Although the mechanism of the association between the SYNTAX score and MACE might be multifactorial, previous reports demonstrated that a higher SYNTAX score was associated with complex CAD and a higher prevalence of diabetes mellitus and peripheral artery disease, suggesting that the SYNTAX score may be related to advanced coronary and systemic atherosclerosis [24, 25].

### Clinical implications

Our study demonstrated that the SYNTAX score is useful for assessing the prognosis of patients with a prior diagnosis of HF undergoing PCI. Given that prior HF patients have a high risk of recurrent cardiovascular events such as sudden death and hospitalization for worsening HF, we recommend that calculation of the SYNTAX score should be performed in prior HF patients with CAD to allow for precise risk stratification for MACE.

### Study limitations

The major limitation of our study is that it was an observational study with a relatively small number of subjects and, therefore, the possibility of selection bias and unmeasured confounding factors might not have been completely excluded. Thus, our results should be interpreted cautiously until verified by large-scale multi-center studies. However, our study is the first to report on the utility of the SYNTAX score in prior HF patients with CAD undergoing PCI. Second, our definition of HF relied on an investigator diagnosis based on the Framingham criteria, rather than a requirement to fulfill all of the criteria recommended in the guidelines. Third, we analyzed the predictive value of SYNTAX score using an individual cutoff point and, therefore, further studies are needed to determine the optimal cutoff points in this patient population. Fourth, we did not assess biomarkers which are known predictors of cardiovascular events, despite the fact that certain novel biomarkers are associated with a higher SYNTAX score [26, 27]. Fifth, only prior HF patients diagnosed with CAD were included in this study and, therefore, our results may not apply to all HF patients. Despite these limitations, our findings provide new insight into the risk stratification for cardiovascular events in prior HF patients undergoing PCI. Furthermore, since our study was based on observational registry data from patients with CAD, we consider that our results represent a real-world unselected population of prior HF patients undergoing PCI.

## Conclusions

In prior HF patients with CAD, high SYNTAX scores predicted a high incidence of MACE. The SYNTAX score might be a useful parameter for improving risk stratification in these patients.
